# The Controversies in the Relationship Between *Helicobacter pylori* Infection and Inflammatory Bowel Disease: Narrative Review

**DOI:** 10.3390/jcm14176083

**Published:** 2025-08-28

**Authors:** Jonatan Vukovic, Ivana Jukic

**Affiliations:** 1 Department of Internal Medicine, Division of Gastroenterology, University Hospital of Split, Spinciceva 1, 21000 Split, Croatia; jonatan.vukovic@mefst.hr; 2 Department of Internal Medicine, School of Medicine, University of Split, Soltanska 2, 21000 Split, Croatia; 3 Faculty of Health Sciences, University of Split, Rudjera Boskovica 35, 21000 Split, Croatia

**Keywords:** *Helicobacter pylori*, inflammatory bowel disease, challenges, eradication, treatment, Crohn’s disease, ulcerative colitis

## Abstract

**Background/Objective:** The relationship between *Helicobacter pylori* (*H. pylori*) infection and inflammatory bowel disease (IBD) remains controversial. While *H. pylori* is a well-established pathogen in gastroduodenal diseases, emerging evidence suggests it may exert immunomodulatory effects that influence the pathogenesis and clinical course of IBD. This review aims to explore the association between *H. pylori* infection and IBD, focusing on infection prevalence among IBD patients, the potential protective or harmful roles of *H. pylori*, and the impact of eradication therapy on IBD onset and activity. **Methods:** A comprehensive literature search was conducted using PubMed up to, including clinical studies, meta-analyses, systematic reviews, and observational data. A total of 40 studies met the inclusion criteria and were critically reviewed. **Results:** The majority of studies indicate a significantly lower prevalence of *H. pylori* infection among patients with IBD compared to the general population. Several meta-analyses support a potential protective effect, particularly in Crohn’s disease and among CagA-positive *H. pylori* strains. However, data on the impact of eradication therapy on IBD progression remain inconclusive. Some studies suggest a higher relapse risk post-eradication, while others report no change in disease activity. Variability in outcomes may be influenced by geographic, demographic, and methodological differences, as well as disease activity at the time of eradication. **Conclusions:** Although numerous studies support an inverse association between *H. pylori* infection and IBD, the nature and direction of this relationship remain unclear. Given the complex interplay between host immunity, gut microbiota, and antibiotic exposure, the decision to eradicate *H. pylori* in IBD patients should be individualized. Further prospective studies are needed to clarify the immunological and microbiological mechanisms underlying this association and to inform clinical guidelines.

## 1. Introduction

*Helicobacter pylori (H. pylori)* has co-evolved with humans for over 60,000 years [1] and was first successfully isolated from a gastric biopsy in 1983 by Marshall and Warren [2]. This spiral-shaped, microaerophilic, Gram-negative bacterium infects more than 50% of the global population [3]. Its prevalence correlates with advancing age and lower socioeconomic status, although substantial geographic and demographic variations exist [4]. Early-life conditions, particularly poor hygiene and overcrowding during childhood, are key risk factors for acquisition [5,6].

*H. pylori* is primarily transmitted via oral–oral and fecal–oral routes, with contaminated water also recognized as a potential source [7]. Infection typically occurs in childhood and, without treatment, persists throughout life [8].

Clinically, *H. pylori* is associated with a wide spectrum of gastrointestinal and extra-gastrointestinal diseases, including peptic ulcer disease, autoimmune gastritis, gastric malignancies such as MALT lymphoma and adenocarcinoma, and extra-digestive conditions like iron deficiency anemia, vitamin B12 deficiency, and idiopathic thrombocytopenic purpura (ITP) [9,10]. Several studies have also noted a higher prevalence of *H. pylori* in patients with autoimmune conditions such as Hashimoto’s thyroiditis, Graves’ disease, systemic lupus erythematosus, rheumatoid arthritis, and primary Sjögren’s syndrome [11].

Interestingly, some studies have reported an inverse association between *H. pylori* colonization and autoimmune diseases, particularly inflammatory bowel diseases (IBD) like Crohn’s disease (CD) and ulcerative colitis (UC), suggesting a possible protective role [12]. While the exact nature of this relationship is unclear, these findings highlight the need for further investigation into potential causality and the clinical relevance of *H. pylori* in autoimmune settings.

IBD is a chronic, relapsing-remitting inflammatory disorder of the gastrointestinal tract, encompassing CD, UC, and related entities [13]. Mucosal inflammation manifests with abdominal pain, diarrhea, rectal bleeding, and weight loss, driven by infiltration of neutrophils and macrophages that release pro-inflammatory mediators [13,14]. The pathogenesis involves a complex interplay of genetic predisposition, environmental triggers (e.g., Westernized diet, smoking, and antibiotic exposure), and immune dysregulation. IBD often includes extraintestinal manifestations such as arthritis, dermatologic disorders, and hepatobiliary involvement [15].

Since the mid-20th century, IBD incidence has increased globally, particularly in industrializing regions of Asia, Africa, and South America, despite stabilization in Western countries [16,17,18]. Prevalence rates are highest in Europe and North America [18], and the burden among the elderly is projected to rise by 2051, posing major public health challenges [19].

Crohn’s disease most often involves the terminal ileum, cecum, and colon in a discontinuous pattern, while ulcerative colitis affects the rectum and extends proximally in a continuous fashion [15,20,21]. CD shows transmural inflammation, fissuring ulcers, and granulomas, while UC is limited to mucosa and submucosa, with features such as cryptitis and crypt abscesses [15,21,22].

The coexistence of *H. pylori* infection and IBD raises important clinical questions. Epidemiological data consistently report lower *H. pylori* prevalence among IBD patients [23], possibly due to antibiotic use, immune dysregulation, or altered gut microbiota. However, the impact of *H. pylori* eradication on IBD remains unclear. Some studies suggest increased relapse risk, while others report no significant effect [23,24,25,26].

Despite ongoing research, the implications of *H. pylori* eradication in IBD are not yet fully understood. Some have proposed that eradication may act as an environmental trigger in genetically susceptible individuals, warranting caution in treatment decisions and highlighting the need for prospective controlled studies. This review explores the complexity of *H. pylori* infection in IBD patients, focusing on prevalence, clinical outcomes, and therapeutic challenges, with the aim of identifying gaps in current knowledge and guiding future research.

## 2. Materials and Methods

### 2.1. Information Source and Search Strategies

The aim of this review was to explore (1) the association between *Helicobacter pylori* (*H. pylori*) infection and inflammatory bowel disease (IBD); (2) the prevalence of *H. pylori* infection in patients with ulcerative colitis and Crohn’s disease; (3) the impact of *H. pylori* eradication therapy on the onset or exacerbation of IBD; and (4) the potential cause-and-effect relationship between these two chronic gastrointestinal conditions. A comprehensive literature search was conducted using the PubMed electronic database from inception to 25 May 2025. PubMed was selected due to its broad coverage of biomedical and clinical research, which aligns with the focus of this review. We acknowledge this as a limitation and note that additional databases, such as Scopus or Web of Science, may contain relevant publications not captured in our search. The search strategy included the use of Medical Subject Headings (MeSH) terms: “*Helicobacter pylori*,” “inflammatory bowel disease,” “eradication,” and “association.” Initially, all terms were combined using the Boolean operator “AND,” but due to limited results, alternative combinations were applied: “*Helicobacter pylori*” AND “inflammatory bowel disease” AND “eradication,” and “*Helicobacter pylori*” AND “inflammatory bowel disease” AND “association.” The reference lists of included studies were also manually screened for additional relevant articles.

Only publications in English and with free full-text access were included. Eligible study types encompassed clinical studies, clinical trials, controlled clinical trials, meta-analyses, multicenter studies, observational studies, randomized controlled trials, reviews, and systematic reviews involving both adult and pediatric populations, as well as animal studies when relevant. Studies were excluded if they met any of the following criteria: (1) primary endpoints not aligned with the aims of this review; (2) case reports, case series, commentaries, letters, preprints, or book chapters; (3) in vitro studies; (4) articles in languages other than English; or (5) publications without full-text availability. All included studies were critically reviewed. A detailed overview of the selected publications is provided in the Results section in tabular format.

### 2.2. Eligibility Criteria

Specific inclusion criteria involved human studies with adult and pediatric study populations and animal studies, too. Clinical studies, clinical trials, controlled clinical trials, meta-analyses, multicenter studies, observational studies, randomized controlled trials, reviews, and systematic reviews written in English with free full text availability are included in our review.

Exclusion criteria for this review were delineated as follows: (1) studies with primary endpoints not aligned with the scope of this review; (2) books and documents, letter, commentaries, preprint, case reports, and case series; (3) in vitro studies; (4) studies published in languages other than English; and (5) articles without full-text availability.

## 3. Results

Using MeSH terms “*Helicobacter pylori*” AND “inflammatory bowel disease” AND “eradication”, the article search identified twenty-six relevant full-text articles from the PubMed electronic database, of which sixteen articles met the full inclusion criteria [12,23,27,28,29,30,31,32,33,34,35,36,37,38,39,40,41]. By searching the electronic database PubMed using MeSH terms “*Helicobacter pylori*” AND “inflammatory bowel disease” AND “association”, we have identified seventy-six full-text articles. After elimination of duplicate articles with the first search and identification of additional manuscripts, twenty-four studies that met the full inclusion criteria for this article were retrieved and fully reviewed [11,42,43,44,45,46,47,48,49,50,51,52,53,54,55,56,57,58,59,60,61,62,63,64,65].

A total of 40 articles were selected for review, including 18 review articles, 9 meta-analyses, 4 systematic reviews, 1 umbrella review of meta-analyses, 2 bibliometric analyses, 3 multicenter studies, and 3 observational studies. The results are presented in Table 1, Table 2 and Table 3.

Research into the relationship between *Helicobacter pylori* infection and inflammatory bowel disease (IBD) began to take shape around 2009 and has gained momentum over the years. Since then, interest in this topic has grown steadily, with a marked increase in studies exploring possible protective, immunological, and microbial mechanisms. Importantly, many of these studies are based on high levels of evidence, including numerous systematic reviews and meta-analyses. These consistently show a lower prevalence of *H. pylori* infection in IBD patients across various populations and settings. The fact that such findings are replicated across different study designs and regions strengthens the validity of this association. Together, the accumulated evidence points to a meaningful biological link that warrants further investigation.

## 4. Discussion

The coexistence of *Helicobacter pylori* infection and Inflammatory Bowel Disease (IBD) presents a unique set of challenges that complicate both diagnosis and management. While *H. pylori* is a recognized pathogen implicated in significant gastric morbidity, its relationship with IBD remains controversial. The lower prevalence of *H. pylori* in IBD patients, consistently reported across multiple studies, raises questions about potential protective roles or interactions between the bacterium and host immunity [11,12,23,31,33,34,35,37,38,39,43,44,46,47,48,49,50,51,52,55,56,57,58,59,60,61,62,63]. This phenomenon has been attributed to several factors, including extensive antibiotic use in IBD management, immune modulation associated with chronic inflammation, and dysbiosis of the gut microbiota. The frequent use of antibiotics, particularly broad-spectrum agents during IBD flares or for perioperative prophylaxis in Crohn’s disease, likely contributes to the observed lower colonization rates. However, this reduced prevalence may not uniformly translate into clinical benefit, as it introduces unique diagnostic and therapeutic challenges.

From a diagnostic perspective, the accuracy of non-invasive tests for *H. pylori*, such as the urea breath test (UBT) and stool antigen test, is diminished in IBD patients due to confounding factors such as active intestinal inflammation and the use of proton pump inhibitors (PPIs). These medications, commonly prescribed for upper GI symptoms in IBD patients, suppress bacterial load and gastric acidity, leading to false-negative test results. Endoscopic biopsy remains the gold standard for diagnosis, offering the opportunity for histological examination and culture, but the invasive nature of this procedure poses risks for IBD patients, particularly during active disease. Moreover, in patients with Crohn’s disease involving the upper GI tract, the differentiation between *H. pylori*-related gastritis and Crohn’s disease-associated gastroduodenitis adds an additional layer of complexity.

Therapeutically, *H. pylori* eradication in IBD patients necessitates a nuanced approach, balancing the benefits of eradication against the potential risks of exacerbating IBD symptoms. Therapy consisting of a PPI, clarithromycin, and amoxicillin achieves eradication rates of approximately 70–80% in the general population. However, rising rates of antibiotic resistance, particularly to clarithromycin and metronidazole, reduce the efficacy of these regimens in IBD patients, many of whom have been exposed to antibiotics for disease management [64]. Bismuth-containing quadruple therapy or regimens tailored based on antibiotic susceptibility testing may be required in these cases.

A critical concern in IBD patients undergoing *H. pylori* eradication therapy is the potential for antibiotic-induced gut microbiota disruption. Dysbiosis, a hallmark of IBD, may be exacerbated by the broad-spectrum antibiotics used in eradication therapy, leading to disease flares or worsening symptoms. Oral supplementation with a narrow spectrum of Gram-positive bacteria has shown clinical improvement in *H. pylori*-related symptoms, yet microbiota alterations may predispose individuals to intestinal or systemic diseases later in life. Emerging evidence suggests that using multi-strain probiotics or paraprobiotics, rather than single-strain formulations, may help reduce the incidence of metabolic disturbances associated with dysbiosis [65].

Considering the diverse effects of different bacterial strains, high-throughput sequencing technologies are essential for characterizing microbiota changes at the strain level and personalizing probiotic interventions. Furthermore, next-generation probiotics and genetically modified microorganisms are being explored to enhance clinical outcomes, either by restoring disease-specific bacterial taxa or by producing therapeutic compounds such as antimicrobial peptides. Innovations such as microencapsulation and nanotechnology are also being developed to optimize probiotic delivery and minimize adverse metabolic effects. These advances may offer promising adjuncts in managing *H. pylori* eradication in the context of IBD [66]. Additionally, PPIs, an essential component of *H. pylori* therapy, may interact with immunosuppressive agents such as azathioprine, methotrexate, or biologics, potentially altering their pharmacokinetics and efficacy. The impact of *H. pylori* eradication on IBD activity remains debated. Some articles suggest that *H. pylori* colonization may exert an immunomodulatory effect by reducing pro-inflammatory cytokine production and promoting regulatory T-cell activity [67,68]. Consequently, eradication might remove this protective mechanism and exacerbate IBD symptoms. Conversely, *H. pylori* may aggravate upper GI symptoms in IBD patients, including dyspepsia or gastric ulceration, thereby impairing quality of life and complicating disease management.

In light of these complexities, a tailored approach to managing *H. pylori* infection in IBD patients is essential. Routine screening may not be justified in asymptomatic individuals, but targeted testing should be performed in patients with upper GI symptoms, a history of peptic ulcer disease, or those requiring long-term PPI use. Whenever possible, eradication therapy should be timed during IBD remission to minimize the risk of flares. A multidisciplinary approach involving gastroenterologists, microbiologists, and clinical pharmacologists is recommended to optimize outcomes and mitigate risks.

Future research should focus on the long-term impact of *H. pylori* eradication on IBD progression, microbiota composition, and patient quality of life. Large-scale studies using germ-free mice colonized with human microbiota, and clinical trials combining broad-spectrum antibiotic regimens with targeted probiotic interventions, are needed to better understand the role of microbiome modulation in this population. The integration of novel diagnostic tools and personalized medicine strategies holds the potential to transform the management of *H. pylori* in the context of IBD and improve clinical outcomes for this unique patient group.

A limitation of this review is the exclusive reliance on the PubMed database for literature retrieval, without inclusion of other major electronic databases such as Web of Science, Scopus, and Cochrane, which may have resulted in the omission of pertinent studies. This limitation underscores the need for future high-quality systematic reviews or meta-analyses based on comprehensive multi-database searches and standardized methodological frameworks to ensure a more exhaustive evaluation of the available evidence.

## 5. Conclusions

While *Helicobacter pylori* eradication offers clear oncological benefits—most notably in the prevention of gastric cancer—it presents distinct challenges in patients with IBD. A key concern is the potential to trigger de novo IBD or exacerbate existing disease following eradication therapy. Although observational studies suggest a possible association, the causal mechanisms remain poorly defined. Current international guidelines lack specific recommendations for *H. pylori* management in IBD patients, underscoring a notable gap in clinical guidance. An often overlooked factor is the disease stage at the time of eradication. Patients in remission may respond differently to microbiota-altering antibiotics than those with active inflammation. The degree of mucosal integrity, immune activity, and dysbiosis likely influences outcomes, making timing critical. Similarly, a patient’s immunosuppressive regimen, prior eradication attempts, and antibiotic resistance patterns may affect therapeutic response and recurrence risk. These factors emphasize the need for an individualized, patient-centered approach rather than standardized treatment strategies. Future studies should stratify patients by disease activity, phenotype, immunosuppressive therapy, and eradication history. Prospective trials with validated clinical, endoscopic, and biochemical endpoints are essential to optimize treatment decisions. In countries with population-wide *H. pylori* screening, guidelines must incorporate considerations specific to IBD—particularly immunological and microbiome-related risks. Clinicians need tools to distinguish true IBD flares from expected post-eradication changes, and decisions to withhold or delay therapy should be based on individualized risk–benefit assessments. Ultimately, managing *H. pylori* in IBD requires a multidisciplinary perspective—integrating gastroenterology, microbiology, and immunology—to support evidence-based, personalized care. This evolving field offers important opportunities to improve patient outcomes and deepen our understanding of host–microbiome interactions in immune-mediated disease.

## 6. Future Directions

Future research should aim to clarify the causal mechanisms underlying the inverse association between *Helicobacter pylori* infection and inflammatory bowel disease, with particular focus on the immunological and microbiome-related pathways involved. It remains important to determine whether the observed relationship reflects a true biological interaction or is influenced by confounding factors such as antibiotic use, disease severity, or treatment history. Further investigation into the role of specific *H. pylori* strains, host genetics, and environmental exposures could provide deeper insight into the variability seen among patients. Additionally, studies assessing the impact of *H. pylori* eradication on IBD activity, therapeutic response, and gut microbial balance will be essential for informed clinical management. Incorporating microbiome analysis, immune profiling, and precision medicine strategies may help identify patient subgroups that could benefit from personalized approaches. A clearer understanding of this complex interplay could ultimately refine both diagnostic and therapeutic practices in IBD care.

## Figures and Tables

**Table 1:** List of studies/research on the topic of the association/eradication between *H. pylori* infection and inflammatory bowel diseases in adult study population.

Authors/Year of Publication (Reference)	Type of Study	Adult Study Population Number/N/ of IBD Subjects or Publications	Results	Conclusions Possible Beneficial Effects of *H. pylori* in IBD YES/NO/UN
Gravina, A.G. et al., 2024. [27]	review	Case reports, N = 5 N = 4 clinical studies, N = 563 subjects	The onset of IBD after the eradication treatment of *H. pylori* infection. No significant association between *H. pylori* eradication and recurrence or exacerbation of IBD	No specific recommendations for this particular situation in the leading international IBD and *H. pylori* guidelines UN
Wang, Z. et al., 2024. [40]	bibliometric analysis	N = 246 publications	The number of papers on *H. pylori* and IBD has increased significantly over the past two decades. China, the United States, and Australia are at the forefront of this field.	Despite notable progress in the last decade, challenges remain. The exact relationship between *H. pylori* and IBD is still uncertain. Many studies suggest that *H. pylori* infection may reduce the risk and severity of IBD, but others present different perspectives. UN
Li, Y. et al., 2024. [41]	bibliometric analysis	N = 1196 publications	Most studies focus on the immune mechanism of *H. pylori* negatively correlated with IBD, and there are still a lot of gaps for researchers to fill. The question of whether *H. pylori* definitively offers protective effects against IBD remains unresolved.	Therefore, further investigation could explore the underlying mechanisms of their relationship or initiate long-term prospective cohort studies to gather more compelling evidence. UN
Feilstrecker Balani, G. et al., 2023. [43]	review	N = UN subjects	*H. pylori* neutrophil-activating protein (HP-NAP) is a virulence factor that plays an important role in immunomodulation.	This review emphasized the role of *H. pylori* CagA+ and HP-NAP in a favorable prognosis of IBD. YES
He, J. et al., 2022. [44]	review	N = UN subjects	Association between CagA seropositivity and lower odds of IBD.	*H. pylori* infection might play a protective role in inflammatory bowel disease (IBD). YES
Abd El-Wahab, E.W. et al., 2022. [45]	prospective observational study	N = 182 subjects	In total, 49.5% patients with IBD had evidence of *H. pylori* infection. The majority of patients who were *H. pylori* positive with IBD had undergone *H. pylori* eradication therapy during the previous 12 months, which raises questions about the efficacy of eradication therapy or reveals reinfection among this group of patients.	The number of patients who recovered from IBD among patients who were *H. pylori* negative was similar to that of patients who were *H. pylori* positive. The association between IBD and *H. pylori* infection is unresolved and should be further investigated. UN
Wang, L. et al., 2022. [11]	review	N = UN subjects	The epidemiological literature generally supports a negative correlation between *H. pylori* and IBD.	Most studies support a negative association between *H. pylori* and IBD, but some scholars suggest that only CagA seropositive *H. pylori* exposure may be relevant to IBD. YES
Axelrad, J.E. et al., 2021. [46]	systematic review	N = 97 studies	Compared with CagA-negative *H. pylori* exposure or *H. pylori* non-exposure overall, exposure to CagA-positive *H. pylori* was associated with a significantly lower odds of IBD.	It is important to emphasize that not all *Helicobacter* species are inversely associated with IBD. YES
Murad, H. et al., 2021. [32]	observational cross-sectional study:	N = 203 subjects	Sequential eradication therapy did not affect serum OPG levels in patients with *H. pylori* infection and co-existing IBD. Thus, serum OPG elevation may be used as a marker of the development of IBD in patients with active or prior *H. pylori* infection.	Further research is recommended. UN
Zhong, Y. et al., 2021. [23]	systematic review	N = 209 studies	IBD, UC, and CD were negatively correlated to *H. pylori* prevalence (all *p* < 0.001). IBD patients were 1.41 times (OR = 1.41, 95% CI = 1.25–1.58) more likely to relapse after eradication of *H. pylori*. Finally, *H. pylori* infection was not related to IBD medication and classification.	*H. pylori* prevalence was negatively correlated with IBD, and *H. pylori* had a protective effect against IBD. Eradication of *H. pylori* can lead to recurrence of IBD. YES
Reshetnyak, V.I. et al., 2021. [33]	review	N = UN subjects	*H. pylori* persistence may be supposed to be a potentially beneficial factor against the development of IBD.	Perform more individualized eradication therapy in the context of assessment of additional risk factors. YES
Gravina, A.G. et al., 2020. [47]	review	N = UN subjects	The severity of IBD, UC in particular, increased after *H. pylori* eradication.	To define whether *H. pylori* products, such as Hp (2–20) peptide, might be considered as potential therapeutic agents in specific clinical settings, such as IBDs. YES
Santos, M.L.C. et al., 2020. [48]	review	N = UN subjects	The composition of gut microbiota, which seems to play a crucial role in IBD development	It is plausible to think that the changes in the intestinal microbiome may be decisive in the IBD onset after *H. pylori* treatment. YES
Axelrad, J.E. et al., 2020. [49]	systematic review	N = 63 studies	*H. pylori* infections were associated with a generally consistent reduced risk of IBD.	*H. pylori* has inverse associations with incident IBD. YES
Imawana, R.A. et al., 2020. [58]	meta-analysis	N = 32 studies N = 4607 IBD subjects	The protective effect of *H. pylori* on IBD varied by both subtype (more protection against CD vs. UC) and region (East Asia more protected than Mediterranean regions).	Protective effect of *H. pylori* against IBD. YES
Pellicano, R. et al., 2020. [59]	review	N = UN	An inverse correlation between H pylori infection and IBD prevalence has been confirmed.	Inverse correlation YES
Tepler, A. et al., 2019. [50]	meta-analysis	N = 3 studies N = 960 subjects	CagA seropositivity was associated with decreased odds of IBD, particularly CD.	We found evidence for a significant association between CagA seropositive *H. pylori* exposure and reduced odds of IBD, particularly CD, but not for CagA seronegative H pylori exposure. YES
Wang, W.L. et al., 2019. [51]	meta-analysis	N = 2055 subjects	There was a significant difference in the Hp infection rate between CD patients and controls, showing a negative correlation.	*H. pylori* infection was negatively associated with the incidence of CD. YES
Piovani, D. et al., 2019. [63]	umbrella review of meta-analyses	N = 53 meta-analysis	*H pylori* infection reduces the risk of IBD (CD, UC, and IBD).	Protective rule of *H. pylori*. YES
Yu, Y. et al., 2018. [35]	review	N = UN subjects	An inverse correlation between *H. pylori* infection and IBD onset. *H. pylori* infection induces tolerogenic dendritic cells and immunosuppressive Tregs, which have a key role in systematic immunomodulation.	The immune tolerance property of *H. pylori* should be thoroughly considered when designing optimized and individualized treatments for *H. pylori*-infected patients. YES
Kayali, S. et al., 2018. [52]	review	N = 22 studies	The difference in prevalence of *H. pylori* infection between IBD-affected patients and controls was significant in 16/22 studies.	Striking inverse association between HP infection and the prevalence of IBD, independently from the type of IBD considered (CD, UC, and IBDU) across distinct geographic regions. YES
Castaño-Rodríguez, N. et al., 2017. [62]	meta-analysis	N = UN subjects	Analyses comprising patients with CD, UC, and IBD showed a consistent negative association between gastric *H. pylori* infection and IBD.	*H. pylori* infection is negatively associated with IBD regardless of ethnicity, age, *H. pylori* detection methods, and previous use of aminosalicylates and corticosteroids. YES
Murad H.A. et al., 2016. [36]	review	N = UN subjects	The present review suggests that measuring fecal calprotectin, and patient counseling and follow-up, on eradicating *H. pylori* in CD patients and/or patients with a high risk for CD, may help monitor CD.	The current data that suggest a positive association between *H. pylori* eradication and development of CD are limited and provide very little evidence. UN
Robinson, K. et al., 2015. [38]	review	N = UN	Significantly reduced risk of IBD when infected with *H. pylori*.	Reduced incidence of *H. pylori* infection in IBD patients. But warned of possible exacerbation following eradication therapy. YES
Wu, X.W. et al., 2015. [60]	meta-analysis	N = 1299 IBD subjects	The *H. pylori* infection rate in Asian IBD patients is significantly lower than in non-IBD patients	Infection protects against the development of IBD. YES
Rokkas, T. et al., 2015. [61]	meta-analysis	N = 4400 IBD subjects	Significant negative association between *H. pylori* infection and IBD	A possible protective benefit of *H. pylori* infection against the development of IBD. YES
Ierardi, E. et al., 2014. [39]	review	N = UN	Low incidence of *H. pylori* infection in patients with IBD compared with normal controls.	The potential protective role of *H. pylori* against inflammatory bowel diseases needs to be better elucidated. YES
Papamichael, K. et al., 2014 [54]	review	N = UN subjects	Potential protective role of *H. pylori* infection against the development of IBD. Rapid onset of CD after eradication of *H. pylori* infection.	The association between *H. pylori* infection and IBD is still controversial; however, it is worthy of further investigation. UN
Xiang, Z. et al., 2013. [57]	retrospective single-center study	N = 229 CD subjects	The *H. pylori* infection rate in the CD group was 27.1%, significantly lower than that of 47.9% in the control group.	Lower *H. pylori* infection in CD patients suggests a correlation between bacterial infection and CD, suggesting caution when considering *H. pylori* eradication in CD patients. YES
Owyang, S.Y. et al., 2012. [55]	review	N = UN subjects	Immunoregulatory properties of the *H. pylori* genome revealed the importance of the TLR-9-mediated mechanism in the pathogenesis of IBD.	*H. pylori* genomic DNA contributes to the beneficial anti-inflammatory effect of *H. pylori* colonization in patients with chronic inflammatory conditions. YES
Luther, J. et al., 2010. [12]	meta-analysis	N = 5903 subjects	In total, 27.1% of IBD patients had evidence of infection with *H. pylori* compared to 40.9% of patients in the control group.	Protective benefit of *H. pylori* infection against the development of IBD. YES
Song, M.J. et al., 2009. [56]	multicenter study	N = 316 subjects	A statistically significant difference in *H. pylori* infection rate was noticed between the IBD patients (25.3%) and the controls (52.5%), and between UC (32.0%) and CD patients (17.7%).	Korean patients with IBD, particularly CD, were found to have a significantly lower *H. pylori* infection rate than the controls. YES

Legends: UN—unknown; *H. pylori—Helicobacter pylori*; *H. pylori*—Helicobacter pylori-infection; IBDs—inflammatory bowel diseases; IBD—inflammatory bowel disease; CAI—clinical activity index; ESS—endoscopic severity score; ET—eradication therapy; FU—follow up; UGE—upper gastroscopy; OPG—osteoprotegerin; UC—ulcerative colitis; CD—Crohn’s disease.

**Table 2:** List of studies/research on the topic of the association/eradication between *H. pylori* infection and inflammatory bowel diseases in pediatric study population.

Authors/Year of Publication (Reference)	Type of Study	Pediatric Study Population Number/N/ of IBD Subjects or Publications	Results	Conclusions Possible Beneficial Effects of *H. pylori* in IBD YES/NO/UN
Dilaghi, E. et al., 2024. [28]	prospective multicenter study	N = 76 subjects	The occurrence of *H. pylori* infection did not differ between IBD and non-IBD patients. No differences in CAI or ESS were observed at the diagnosis, and after ET, no worsening of CAI or ESS was noted at one-year FU, between *H. pylori*-positive and -negative IBD patients.	No association between *H. pylori* eradication and exacerbation of IBD. NO
Kotilea, K. et al., 2024. [29]	retrospective multicenter study	N = 1292 subjects	*H. pylori* was identified in 8.5% IBD patients. The prevalence differed significantly between Europe (Eastern 5.2%, Southern 3.8%, Western 5.6%) and the Middle East 26.6%. Eradication treatment was prescribed in 35.8% of IBD cases.	Identifying *H. pylori* incidentally during UGE performed for the most common gastrointestinal diseases varies significantly among regions but not among diseases. NO
Kong, G. et al., 2023. [42]	meta-analysis	N = 2236 subjects	No significant difference in *H. pylori* prevalence (9.8% vs. 12.7%) by comparing the children IBD group to controls. In children suffering from UC and CD, the *H. pylori* infection rates were higher than in those with IBD-unclassified.	No correlation was found between *H. pylori* infection and the occurrence of IBD in children. NO
Ravikumara M., 2023. [31]	review	N = UN subjects	*H. pylori* is a potent modulator of the immune system and prevents IBD.	Possible beneficial effects *H. pylori* may confer against IBD, especially in childhood. YES
Aguilera Matos, I. et al., 2020. [34]	review	N = UN subjects	Meta-analysis suggests a strong inverse association with CD in children.	*H. pylori* may have immunoregulatory properties in IBD, and the inverse association seems stronger in pediatric patients and those with CD. YES

Legends: UN—unknown; *H. pylori—Helicobacter pylori*; *H. pylori*—Helicobacter pylori-infection; IBDs—inflammatory bowel diseases; IBD—inflammatory bowel disease; CAI—clinical activity index; ESS—endoscopic severity score; ET—eradication therapy; FU—follow up; UGE—upper gastroscopy; OPG—osteoprotegerin; UC—ulcerative colitis; CD—Crohn’s disease.

**Table 3:** List of studies/research on the topic of the association/eradication between *H. pylori* infection and inflammatory bowel diseases in pediatric and adult study populations.

Authors/Year of Publication (Reference)	Type of Study	Pediatric and Adult Study Population Number/N/ of IBD Subjects or Publications	Results	Conclusions Possible Beneficial Effects of *H. pylori* in IBD YES/NO/UN
Bretto, E. et al., 2024. [30]	systematic review	N = more than 6000 subjects	Reduced incidence of *H. pylori* infection in patients with IBDs. Potential protective role of *H. pylori* against the development of immune-mediated diseases, particularly when considering the CagA-positive strain, regardless of age, ethnicity, and previous treatment with corticosteroids, antibiotics, and mesalazine. Conflicting findings highlight potential risks, particularly in CD.	The relationship between *H. pylori* infection and IBDs remains a topic of debate, with conflicting evidence from different studies. UN
Arnold, I.C. et al., 2016. [37]	review	N = UN subjects	*H. pylori* infection is inversely associated with both CD and UC in European, Asian, and American populations. Inverse association is especially strong in CD patients, children, and young adults. *H. pylori* reduces clinical and histopathological IBD symptoms.	*H. pylori* is inversely associated with, and likely protective against, IBDs. YES
Yu, Q. et al., 2015. [53]	meta-analysis	N = 14 studies (11 adult studies and 3 pediatric studies) N = 739 subjects	The patients with IBD tended to have a higher prevalence of enterohepatic Helicobacter species in the intestinal mucosa, although the prevalence of *H. pylori* was not significantly higher.	It appears that enterohepatic Helicobacter species was associated with IBD, while intestinal *H. pylori* infection was not significantly associated with IBD. NO

Legends: UN—unknown; *H. pylori—Helicobacter pylori*; *H. pylori*—Helicobacter pylori-infection; IBDs—inflammatory bowel diseases; IBD—inflammatory bowel disease; CAI—clinical activity index; ESS—endoscopic severity score; ET—eradication therapy; FU—follow up; UGE—upper gastroscopy; OPG—osteoprotegerin; UC—ulcerative colitis; CD—Crohn’s disease.

## References

[1] Moodley Y., Linz B., Bond R.P., Nieuwoudt M., Soodyall H., Schlebusch C.M., Bernhöft S., Hale J., Suerbaum S., Mugisha L. (2012). Age of the association between Helicobacter pylori and man. PLoS Pathog..

[2] Warren J.R., Marshall B. (1983). Unidentified curved bacilli on gastric epithelium in active chronic gastritis. Lancet.

[3] Hooi J.K.Y., Lai W.Y., Ng W.K., Suen M.M.Y., Underwood F.E., Tanyingoh D., Malfertheiner P., Graham D.Y., Wong V.W.S., Wu J.C.Y. (2017). Global Prevalence of Helicobacter pylori Infection: Systematic Review and Meta-Analysis. Gastroenterology.

[4] Azevedo N.F., Huntington J., Goodman K.J. (2009). The epidemiology of Helicobacter pylori and public health implications. Helicobacter.

[5] Eusebi L.H., Zagari R.M., Bazzoli F. (2014). Epidemiology of Helicobacter pylori infection. Helicobacter.

[6] Nguyen J., Kotilea K., Bontems P., Miendje Deyi V.Y. (2023). *Helicobacter pylori* Infections in Children. Antibiotics.

[7] Brown L.M. (2000). Helicobacter pylori: Epidemiology and routes of transmission. Epidemiol. Rev..

[8] Wroblewski L.E., Peek R.M. (2021). Helicobacter pylori: A stealth assassin. Trends Cancer.

[9] Wong F., Rayner-Hartley E., Byrne M.F. (2014). Extraintestinal manifestations of Helicobacter pylori: A concise review. World J. Gastroenterol..

[10] Malfertheiner P., Megraud F., O’Morain C.A., Atherton J., Axon A.T., Bazzoli F., Gensini G.F., Gisbert J.P., Graham D.Y., Rokkas T. (2012). European Helicobacter Study Group. Management of Helicobacter pylori infection—The Maastricht IV/ Florence Consensus Report. Gut.

[11] Wang L., Cao Z.M., Zhang L.L., Dai X.C., Liu Z.J., Zeng Y., Li X.Y., Wu Q.J., Lv W.L. (2022). *Helicobacter Pylori* and Autoimmune Diseases: Involving Multiple Systems. Front. Immunol..

[12] Luther J., Dave M., Higgins P.D., Kao J.Y. (2010). Association between Helicobacter pylori infection and inflammatory bowel disease: A meta-analysis and systematic review of the literature. Inflamm. Bowel Dis..

[13] Szigethy E., McLafferty L., Goyal A. (2010). Inflammatory bowel disease. Child Adolesc. Psychiatr. Clin. N. Am..

[14] Stokkers P.C., Hommes D.W. (2004). New cytokine therapeutics for inflammatory bowel disease. Cytokine.

[15] Khor B., Gardet A., Xavier R.J. (2011). Genetics and pathogenesis of inflammatory bowel disease. Nature.

[16] Korzenik J.R., Podolsky D.K. (2006). Evolving knowledge and therapy of inflammatory bowel disease. Nat. Rev. Drug Discov..

[17] Hanauer S.B. (2006). Inflammatory bowel disease: Epidemiology, pathogenesis, and therapeutic opportunities. Inflamm. Bowel Dis..

[18] Ng S.C., Shi H.Y., Hamidi N., Underwood F.E., Tang W., Benchimol E.I., Panaccione R., Ghosh S., Wu J.C.Y., Chan F.K.L. (2017). Worldwide incidence and prevalence of inflammatory bowel disease in the 21st century: A systematic review of population-based studies. Lancet.

[19] Liu Y., Li J., Yang G., Meng D., Long X., Wang K. (2024). Global burden of inflammatory bowel disease in the elderly: Trends from 1990 to 2021 and projections to 2051. Front. Aging.

[20] Yamamoto S., Ma X. (2009). Role of Nod2 in the development of Crohn’s disease. Microbes Infect..

[21] Abraham C., Cho J.H., Cho J.H. (2009). Inflammatory bowel disease. N. Engl. J. Med..

[22] Gajendran M., Loganathan P., Catinella A.P., Hashash J.G. (2018). A comprehensive review and update on Crohn’s disease. Dis. Mon..

[23] Zhong Y., Zhang Z., Lin Y., Wu L. (2021). The Relationship Between Helicobacter Pylori and Inflammatory Bowel Disease. Arch. Iran. Med..

[24] Rosania R., Von Arnim U., Link A., Rajilic-Stojanovic M., Franck C., Canbay A., Malfertheiner P., Venerito M. (2018). Helicobacter Pylori Eradication Therapy Is Not Associated with the Onset of Inflammatory Bowel Diseases. A Case-Control Study. J. Gastrointestin Liver Dis..

[25] Shinzaki S., Fujii T., Bamba S., Ogawa M., Kobayashi T., Oshita M., Tanaka H., Ozeki K., Takahashi S., Kitamoto H. (2018). Seven Days Triple Therapy for Eradication of Helicobacter Pylori Does Not Alter the Disease Activity of Patients with Inflammatory Bowel Disease. Intest. Res..

[26] Lahat A., Kopylov U., Neuman S., Levhar N., Yablecovitch D., Avidan B., Weiss B., Ben-Horin S., Eliakim R., on behalf of the Israeli IBD research Network (IIRN) (2017). Helicobacter Pylori Prevalence and Clinical Significance in Patients with Quiescent Crohn’s Disease. BMC Gastroenterol..

[27] Gravina A.G., Pellegrino R., Iascone V., Palladino G., Federico A., Zagari R.M. (2024). Impact of *Helicobacter pylori* Eradication on Inflammatory Bowel Disease Onset and Disease Activity: To Eradicate or Not to Eradicate?. Diseases.

[28] Dilaghi E., Felici E., Lahner E., Pilozzi E., Furio S., Lucchini L., Quatrale G., Piccirillo M., Parisi P., Curto S. (2024). Helicobacter Pylori infection in children with inflammatory bowel disease: A prospective multicenter study. BMC Pediatr..

[29] Kotilea K., Romano C., Miele E., Kindermann A., Dolstra Y., Misak Z., Urbonas V., Sykora J., Urruzuno P., Krauthammer A. (2024). ESPGHAN *H. pylori* special interest group. Helicobacter pylori infection found during upper endoscopy performed for the diagnosis of celiac, inflammatory bowel diseases, and eosinophilic esophagitis: A multicenter pediatric European study. Helicobacter.

[30] Bretto E., Frara S., Armandi A., Caviglia G.P., Saracco G.M., Bugianesi E., Pitoni D., Ribaldone D.G. (2024). *Helicobacter pylori* in Inflammatory Bowel Diseases: Active Protagonist or Innocent Bystander?. Antibiotics.

[31] Ravikumara M. (2023). *Helicobacter pylori* in children: Think before you kill the bug!. Therap. Adv. Gastroenterol..

[32] Murad H., Rafeeq M., Mosli M., Gari M., Basheikh M. (2021). Effect of sequential eradication therapy on serum osteoprotegerin levels in patients with *Helicobacter pylori* infection and co-existing inflammatory bowel disease. J. Int. Med. Res..

[33] Reshetnyak V.I., Burmistrov A.I., Maev I.V. (2021). *Helicobacter pylori*: Commensal, symbiont or pathogen?. World J. Gastroenterol..

[34] Aguilera Matos I., Diaz Oliva S.E., Escobedo A.A., Villa Jiménez O.M., Velazco Villaurrutia Y.D.C. (2020). *Helicobacter pylori* infection in children. BMJ Paediatr. Open..

[35] Yu Y., Zhu S., Li P., Min L., Zhang S. (2018). Helicobacter pylori infection and inflammatory bowel disease: A crosstalk between upper and lower digestive tract. Cell Death Dis..

[36] Murad H.A. (2016). Does Helicobacter pylori eradication therapy trigger or protect against Crohn’s disease?. Acta Gastroenterol. Belg..

[37] Arnold I.C., Müller A. (2016). *Helicobacter pylori*: Does Gastritis Prevent Colitis?. Inflamm. Intest. Dis..

[38] Robinson K. (2015). *Helicobacter pylori*-Mediated Protection against Extra-Gastric Immune and Inflammatory Disorders: The Evidence and Controversies. Diseases.

[39] Ierardi E., Goni E., Losurdo G., Di Mario F. (2014). Helicobacter pylori and nonmalignant diseases. Helicobacter.

[40] Wang Z., Zhao S., Zhong X., Su Y., Song Y., Li J., Shi Y. (2024). Debate on the relationship between *Helicobacter pylori* infection and inflammatory bowel disease: A bibliometric analysis. Front. Microbiol..

[41] Li Y., Li L., Yin W., Wan J., Zhong X. (2024). Bibliometric analysis of the correlation between *H. pylori* and inflammatory bowel disease. JGH Open.

[42] Kong G., Liu Z., Lu Y., Li M., Guo H. (2023). The association between Helicobacter pylori infection and inflammatory bowel disease in children: A systematic review with meta-analysis. Medicine.

[43] Feilstrecker Balani G., Dos Santos Cortez M., Picasky da Silveira Freitas J.E., Freire de Melo F., Zarpelon-Schutz A.C., Teixeira K.N. (2023). Immune response modulation in inflammatory bowel diseases by *Helicobacter pylori* infection. World J. Gastroenterol..

[44] He J., Liu Y., Ouyang Q., Li R., Li J., Chen W., Hu W., He L., Bao Q., Li P. (2022). *Helicobacter pylori* and unignorable extragastric diseases: Mechanism and implications. Front. Microbiol..

[45] Abd El-Wahab E.W., Youssef E.I., Hassouna E. (2022). *Helicobacter pylori* infection in patients with inflammatory bowel diseases: A single-centre, prospective, observational study in Egypt. BMJ Open.

[46] Axelrad J.E., Cadwell K.H., Colombel J.F., Shah S.C. (2021). The role of gastrointestinal pathogens in inflammatory bowel disease: A systematic review. Therap. Adv. Gastroenterol..

[47] Gravina A.G., Priadko K., Ciamarra P., Granata L., Facchiano A., Miranda A., Dallio M., Federico A., Romano M. (2020). Extra-Gastric Manifestations of *Helicobacter pylori* Infection. J. Clin. Med..

[48] Santos M.L.C., de Brito B.B., da Silva F.A.F., Sampaio M.M., Marques H.S., Oliveira E Silva N., de Magalhães Queiroz D.M., de Melo F.F. (2020). *Helicobacter pylori* infection: Beyond gastric manifestations. World. J. Gastroenterol..

[49] Axelrad J.E., Cadwell K.H., Colombel J.F., Shah S.C. (2020). Systematic review: Gastrointestinal infection and incident inflammatory bowel disease. Aliment. Pharmacol. Ther..

[50] Tepler A., Narula N., Peek R.M., Patel A., Edelson C., Colombel J.F., Shah S.C. (2019). Systematic review with meta-analysis: Association between Helicobacter pylori CagA seropositivity and odds of inflammatory bowel disease. Aliment. Pharmacol. Ther..

[51] Wang W.L., Xu X.J. (2019). Correlation Between Helicobacter Pylori Infection and Crohn’s Disease: A Meta-Analysis. Eur. Rev. Med. Pharmacol. Sci..

[52] Kayali S., Gaiani F., Manfredi M., Minelli R., Nervi G., Nouvenne A., Leandro G., Di Mario F., De’ Angelis G.L. (2018). Inverse association between Helicobacter pylori and inflammatory bowel disease: Myth or fact?. Acta Biomed..

[53] Yu Q., Zhang S., Li L., Xiong L., Chao K., Zhong B., Li Y., Wang H., Chen M. (2015). Enterohepatic Helicobacter Species as a Potential Causative Factor in Inflammatory Bowel Disease: A Meta-Analysis. Medicine.

[54] Papamichael K., Konstantopoulos P., Mantzaris G.J. (2014). Helicobacter pylori infection and inflammatory bowel disease: Is there a link?. World J. Gastroenterol..

[55] Owyang S.Y., Luther J., Owyang C.C., Zhang M., Kao J.Y. (2012). Helicobacter pylori DNA’s anti-inflammatory effect on experimental colitis. Gut Microbes.

[56] Song M.J., Park D.I., Hwang S.J., Kim E.R., Kim Y.H., Jang B.I., Lee S.H., Ji J.S., Shin S.J. (2009). The prevalence of Helicobacter pylori infection in Korean patients with inflammatory bowel disease, a multicenter study. Korean J. Gastroenterol..

[57] Xiang Z., Chen Y.P., Ye Y.F., Ma K.F., Chen S.H., Zheng L., Yang Y.D., Jin X. (2013). Helicobacter pylori and Crohn’s disease: A retrospective single-center study from China. World J. Gastroenterol..

[58] Imawana R.A., Smith D.R., Goodson M.L. (2020). The relationship between inflammatory bowel disease and Helicobacter pylori across East Asian, European and Mediterranean countries: A meta-analysis. Ann. Gastroenterol..

[59] Pellicano R., Ianiro G., Fagoonee S., Settanni C.R., Gasbarrini A. (2020). Review: Extragastric diseases and Helicobacter pylori. Helicobacter.

[60] Wu X.-W., Ji H.-Z., Yang M.-F., Wu L., Wang F.Y. (2015). Helicobacter pylori infection and inflammatory bowel disease in Asians: A meta-analysis. World J. Gastroenterol..

[61] Rokkas T., Gisbert J.P., Niv Y., O’Morain C. (2015). The association between Helicobacter pylori infection and inflammatory bowel disease based on meta-analysis. United Eur. Gastroenterol. J..

[62] Castaño-Rodríguez N., Kaakoush N.O., Lee W.S., Mitchell H.M. (2017). Dual role of Helicobacter and Campylobacter species in IBD: A systematic review and meta-analysis. Gut.

[63] Piovani D., Danese S., Peyrin-Biroulet L., Nikolopoulos G.K., Lytras T., Bonovas S. (2019). Environmental Risk Factors for Inflammatory Bowel Diseases: An Umbrella Review of Meta-analyses. Gastroenterology.

[64] Matsumoto H., Shiotani A., Graham D.Y. (2019). Current and Future Treatment of Helicobacter Pylori Infections. Adv. Exp. Med. Biol..

[65] Nabavi-Rad A., Sadeghi A., Asadzadeh Aghdaei H., Yadegar A., Smith S.M., Zali M.R. (2022). The double-edged sword of probiotic supplementation on gut microbiota structure in *Helicobacter pylori* management. Gut Microbes.

[66] Aijun X., Mengge G., Haishun D., Xuejun P. (2024). Next-generation probiotics delivery: Innovations and applications of single-cell encapsulation. Curr. Opin. Food Sci..

[67] Bartels L.E., Dahlerup J.F. (2017). Association of *Helicobacter pylori* and Crohn’s Disease Incidence: An Inversion Reaction?. Dig. Dis. Sci..

[68] Alotaibi A.D., Al-Abdulwahab A.A., Ismail M.H., AlElyani J.M., Alamri T.A., Alsulaiman R.M., Alhafid I.A., Alzahrani I.M., AlSulaiman R.S., Althubaity A. (2025). Prevalence of H. Pylori in inflammatory bowel disease patients and its association with severity. BMC Gastroenterol..

